# Overlap Syndrome Between Autoimmune Hepatitis and Primary Sclerosing Cholangitis in a Seven-Year-Old Child: A Case Report

**DOI:** 10.7759/cureus.109381

**Published:** 2026-05-21

**Authors:** Chaymae Cherrabi, Amal Hamami, Abdeladim Babakhouya, Imane Kamaoui, Maria Rkain

**Affiliations:** 1 Department of Pediatrics, Centre Hospitalier Universitaire Mohammed VI, Oujda, MAR; 2 Department of Pediatrics, Mohammed VI University Hospital, Faculty of Medicine and Pharmacy, Mohammed First University, Oujda, MAR; 3 Department of Radiology, Mohammed VI University Hospital, Faculty of Medicine and Pharmacy, Mohammed First University, Oujda, MAR; 4 Department of Pediatric Gastroenterology, CHU Mohammed VI Oujda, Oujda, MAR

**Keywords:** autoimmune hepatitis, child, immunosuppression, overlap syndrome, primary sclerosing cholangitis

## Abstract

Overlap syndrome between autoimmune hepatitis (AIH) and primary sclerosing cholangitis (PSC) is a rare condition in children and represents a diagnostic challenge due to the lack of standardized diagnostic criteria and the frequent presence of atypical clinical manifestations. We report the case of a seven-year-old girl presenting with chronic jaundice, severe hepatocellular cytolysis, polyclonal hypergammaglobulinemia, and biliary abnormalities detected on MRI. Liver histology confirmed AIH. The diagnosis of AIH-PSC overlap syndrome was established, and treatment with prednisone and azathioprine was initiated, resulting in significant clinical and biochemical improvement. This case highlights the importance of early diagnostic suspicion and multidisciplinary management in children presenting with complex autoimmune liver disease.

## Introduction

Autoimmune hepatitis (AIH) is an inflammatory disease of the liver that develops in childhood and adulthood. The cause of AIH has not yet been identified. However, the disease predominantly affects females. The clinical presentation of the disorder may vary widely from subclinical manifestations to the occurrence of acute liver failure and complications associated with cirrhosis. Diagnosis is based on elevated serum IgG levels, the presence of autoantibodies, and inflammatory changes on liver biopsy [1]. AIH is classified into two subtypes: type 1, more common in adolescents and associated with antinuclear antibody (ANA)/anti-smooth muscle antibody (ASMA) positivity, and type 2, more frequent in younger children and associated with anti-liver kidney microsomal type 1 (LKM1) antibodies and stronger female predominance [1].

 Primary sclerosing cholangitis (PSC) refers to a chronic cholestatic disease characterized by progressive inflammation and fibrosis of the intrahepatic and extrahepatic bile ducts. The prevalence and incidence of PSC in Northern Europe have been estimated at approximately 1 per 10,000 and 1 per 100,000 per year, respectively. The exact etiology of PSC remains unknown. In patients presenting with a cholestatic biochemical profile, the diagnosis of large-duct PSC is established in the presence of typical cholangiographic features of sclerosing cholangitis on high-quality imaging, after exclusion of secondary causes. Magnetic resonance cholangiopancreatography (MRCP) is considered the preferred non-invasive diagnostic modality [2].

Overlap syndromes refer to the coexistence of more than one autoimmune disease. AIH-PSC is a rare entity characterized by the coexistence of biochemical and histological features of AIH together with cholangiographic abnormalities consistent with PSC. Diagnosing this condition remains problematic due to the lack of universally accepted diagnostic criteria [3].

## Case presentation

We report the case of a seven-year-and-six-month-old girl, the second child of a family of three, born from a third-degree consanguineous marriage, who was admitted for the evaluation of a two-month history of progressive jaundice associated with pruritus, asthenia, and no fever. Her antenatal and perinatal history was unremarkable. She was born at term via vaginal delivery, had normal growth and development, and her vaccinations were up to date according to the national immunization schedule.

On physical examination, the patient was conscious, afebrile, and hemodynamically and respiratory stable. She had marked cutaneous and mucosal jaundice. Abdominal examination revealed a soft but distended abdomen with hepatomegaly measuring approximately five fingerbreadths below the right costal margin, without splenomegaly. The remaining physical examination was normal.

Laboratory investigations showed severe hepatocellular injury with aspartate aminotransferase at 3957 IU/L and alanine aminotransferase at 1909 IU/L, associated with moderate cholestasis (gamma-glutamyl transpeptidase: 107 IU/L and alkaline phosphatase: 369 IU/L). Total bilirubin was 175 mg/L (direct bilirubin: 139 mg/L), and lactate dehydrogenase was elevated at 908 IU/L. International normalized ratio was 1.4, with albumin at 37 g/L. A marked polyclonal hypergammaglobulinemia was present, with elevated IgG at 17.59 g/L (Table 1). Direct Coombs test was positive (IgG 2+). Viral serologies were negative. Classical autoantibodies (ANA, ASMA, anti-LKM1, anti-liver cytosol antibodies, and anti-asialoglycoprotein receptor antibodies) were absent, while anti-double-stranded DNA antibodies were positive. Twenty-four-hour urine protein was negative, and renal function tests and thyroid profile were normal. No clinical or biological criteria suggestive of systemic lupus erythematosus or autoimmune polyendocrine syndrome type 1 were identified. Screening for inflammatory bowel disease was negative, with no digestive symptoms and normal fecal calprotectin levels.

**Table 1:** Evolution of laboratory parameters ALP: Alkaline phosphatase; ALT: Alanine aminotransferase; AST: Aspartate aminotransferase; GGT: Gamma-glutamyl transpeptidase.

Analyte	At admission	Day 7 of steroids	6 weeks of steroids	3 months of steroids	Reference range
AST (U/L)	3957	461	30	24	5-34 UI/L
ALT (U/L)	1909	698	41	14	0-55 UI/L
ALP (U/L)	369	267	149	310	<500 UI/L
GGT(U/L)	908	191	39	29	9-36 UI/L
Total bilirubin (mg/L)	175	53	5.81	3.5	2-12 mg/L
Direct bilirubin (mg/L)	139	43	3.47	2.4	0-5 mg/L

From a phenotypic perspective, the initial presentation was suggestive of AIH, given the marked hepatocellular injury, elevated IgG, and autoimmune cytopenic features.

Abdominal ultrasound showed homogeneous hepatomegaly. MRCP, together with axial contrast-enhanced MRI findings, confirmed the diagnosis of PSC with intra- and extrahepatic bile duct involvement, demonstrating enhancement and irregular thickening of the proximal bile ducts associated with peripheral rarefaction of the intrahepatic bile ducts, resulting in a "dead tree appearance" (Figure 1), as well as a characteristic "beaded" appearance of the intrahepatic bile ducts (Figure 2). Overall, imaging findings supported a cholangiopathic process consistent with PSC.

**Figure 1:**
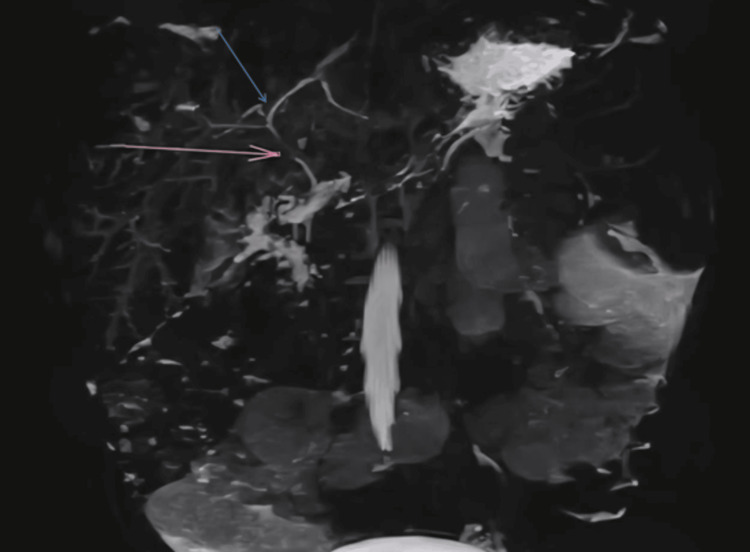
Axial contrast-enhanced MRI showing proximal bile duct enhancement and intrahepatic duct rarefaction Axial contrast-enhanced MRI demonstrating enhancement and irregular thickening of the proximal bile ducts (pink arrow) with peripheral rarefaction of the intrahepatic bile ducts, giving a “dead tree appearance” (blue arrow).

**Figure 2:**
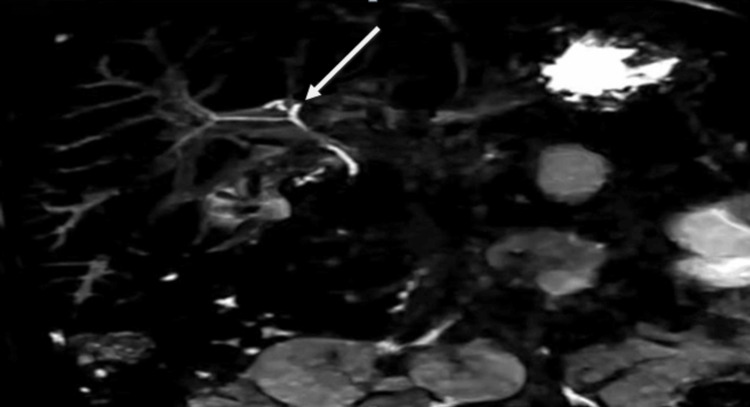
MRI findings in primary sclerosing cholangitis “Beaded” appearance (arrow) of intrahepatic bile ducts is a characteristic of sclerosing cholangitis.

Liver biopsy revealed chronic hepatitis graded A2F2, with moderate interface hepatitis and hepatocyte rosette formation, findings consistent with AIH and supporting an AIH-predominant component of the disease (Figures 3-5).

**Figure 3:**
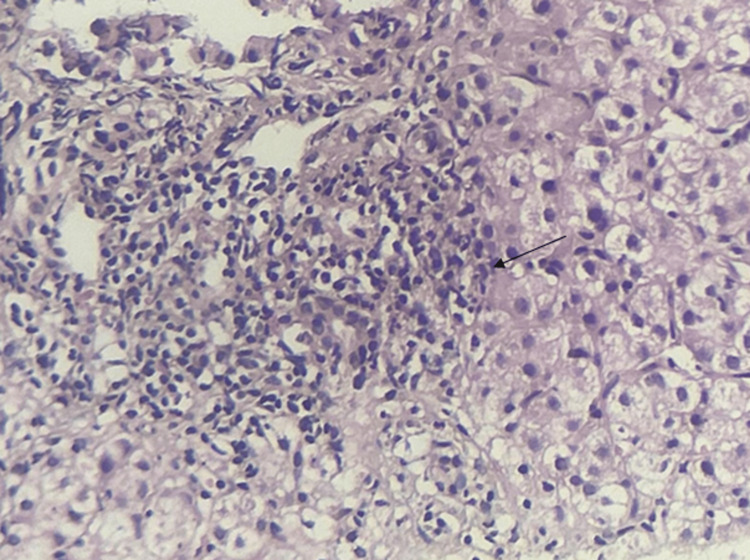
Histopathological findings of autoimmune hepatitis Liver biopsy (hematoxylin and eosin, ×20) showing portal tract with subacute inflammatory infiltrate (arrow) and mild interface hepatitis.

**Figure 4:**
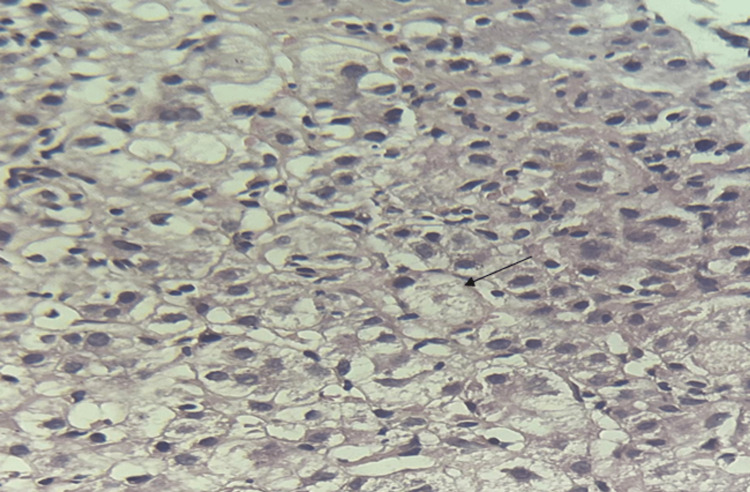
Liver biopsy showing interface hepatitis Liver biopsy (hematoxylin and eosin, ×20) demonstrating features of autoimmune hepatitis, including ballooned hepatocytes (arrow) and hepatocyte rosette formation.

**Figure 5:**
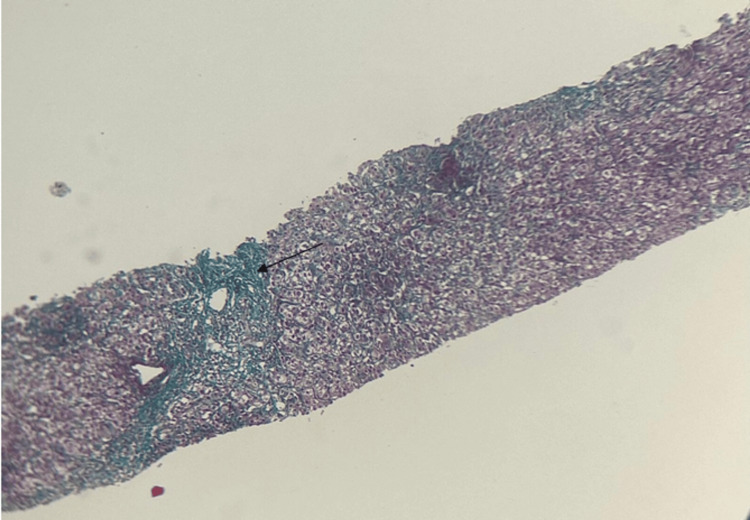
Portal inflammatory infiltrate in autoimmune hepatitis Liver biopsy (trichrome stain) showing periportal fibrosis with septa formation (arrow), consistent with stage F2 fibrosis.

The diagnosis of overlap syndrome between AIH and PSC was established, and treatment with prednisone (2 mg/kg/day) and azathioprine was initiated. Azathioprine was progressively increased from 0.5 mg/kg/day to 2 mg/kg/day and is currently maintained as long-term immunosuppressive therapy. Regular clinical and hematological monitoring was performed, with no adverse effects observed during follow-up. Thiopurine methyltransferase testing was not available before treatment initiation. Ursodeoxycholic acid (UDCA) was not initially available and was not introduced later because of the favorable regression of cholestatic parameters under corticosteroid therapy. Clinical evolution was favorable, with complete resolution of jaundice, clinical improvement, and progressive normalization of liver function tests (Table 1). After nearly one year of follow-up, the patient remains stable under treatment with ongoing immunosuppression and gradual tapering of corticosteroids.

## Discussion

The presence of overlap syndrome between AIH and PSC in children is still uncommon, and the diagnosis is difficult due to a lack of diagnostic criteria. Patients may present with clinical symptoms and biological signs characteristic of isolated AIH with significant cytolysis, hypergammaglobulinemia, and interface hepatitis on histology, as observed in our patient presenting with marked hepatocyte damage and a high level of IgG. In addition, diagnosis could be delayed, especially in the presence of absent classical autoantibodies, which are frequently observed in children [1-3]. The absence of classical autoantibodies in our case also made the differential diagnosis more complicated.

Overlap syndromes are observed in about 3% to 7% of adult patients with autoimmune liver disease [4]. Among primary biliary cholangitis patients, there is 2% to 19% incidence of autoimmunity of hepatitis [5]. In pediatric patients, according to a prospective study conducted by Kumar et al., 10.5% of the AIH group had features of ASC, defined as the pediatric counterpart of AIH-PSC overlap syndrome [6]. Our patient is also consistent with this pediatric population age-wise.

Clinical signs of overlap syndrome are characterized on a case-by-case basis, depending on the underlying dominant autoimmune condition. According to the retrospective study conducted by Heurgué et al., out of the 115 patients with autoimmune liver conditions, jaundice (20%) and pruritus (20%) were the most common symptoms observed in the overlap syndrome among patients with AIH and primary biliary cirrhosis. At the biochemical level, the group with overlap syndrome had higher transaminases and gammaglobulin levels than those observed in isolated PBC cases. On the other hand, their alkaline phosphatase, gamma-glutamyl transpeptidase, and IgM were also higher than in isolated AIH, suggesting the presence of both conditions [7].

The radiological characteristics of AIH-PSC overlap syndrome depend on the dominant component. The PSC-dominant type (PSC predominance) appears on MRI as: central macronodularity, peripheral atrophy, “beaded” appearance of the bile ducts, and biliary dilatation [8]. The presence of one of the first three abnormalities is highly specific for this type, as illustrated in our case by the identification of a “beaded” appearance of the intra- and extrahepatic bile ducts. In contrast, the non-PSC type (AIH predominance) shows relatively normal liver morphology and minimal biliary abnormalities [8].

Histological assessment is an important tool in diagnosing overlap syndromes of AIH and PSC. The typical histological picture usually includes elements from both conditions. There is evidence of interface hepatitis, along with an abundant lymphoplasmacytic infiltrate, which represents the AIH side. Additionally, some elements suggest PSC, such as peri-ductular fibrosis, concentrically arranged ("onion skin") fibrosis, and proliferation or distortion of small bile ducts. Other overlapping elements include irregular canalicular injuries, mixed portal inflammation, and hepatocellular rosettes, which indicate autoimmune inflammatory reactions. The presence of these elements can aid in diagnosing these conditions simultaneously if clinical or biochemical factors point to them [9].

The diagnosis of AIH-PSC overlap syndrome in our patient was established based on a combination of clinical, biochemical, radiological, and histological findings. The presence of marked hepatocellular injury, elevated serum IgG levels, and interface hepatitis on liver biopsy fulfilled the diagnostic features suggestive of AIH. In parallel, cholestatic enzyme elevation together with typical cholangiographic abnormalities on MRCP, including multifocal strictures with a “beaded” appearance of intra- and extrahepatic bile ducts, were consistent with PSC. The coexistence of these hepatitic and cholangiopathic features supported the diagnosis of overlap syndrome (PSC-dominant).

Due to the rarity of overlap AIH/PSC syndrome, management has yet to be standardized because of difficulties in conducting controlled trials. The available data mostly consist of retrospective series and clinical observation [10,11]. Our patient responded positively to immunosuppressive treatment involving corticosteroids and azathioprine, with quick normalization of biochemical tests and absence of yellowing of the skin. For PSC management, UDCA at the rate of 13-15 mg/kg/day is used; for AIH, immunosuppression using corticosteroids, azathioprine, mycophenolate mofetil, or tacrolimus is recommended [1,12]. While UDCA therapy works in some cases, in the majority of patients, the combination of the two therapies is necessary [10,11]. According to a trial by Chazouillères et al. on 17 patients, combination therapy is also more effective in reducing the development of fibrosis than UDCA therapy alone [10]. In advanced cases, liver transplantation is the only way to effectively treat overlap syndrome and provides survival of about 90% after 1 year and 80% at 10 years [13].

In terms of the prognosis, one study of 16 patients with an overlap syndrome of AIH/PSC demonstrated that while biological parameters were improved due to the use of immunosuppression, there was still progression of fibrosis even under medication after a median follow-up time of 12 years. By the end of this period, 12 out of 16 patients had acquired cirrhosis, while 3 had also acquired complications of cirrhosis. This information reveals that the prognosis for this condition is largely dependent upon the progression of hepatic fibrosis despite the administration of suitable medication [14].

## Conclusions

In conclusion, this case highlights the diagnostic challenge of pediatric AIH-PSC overlap syndrome due to its heterogeneous presentation and the absence of standardized diagnostic criteria. The diagnosis was established based on a combination of clinical, laboratory, radiological, and histopathological findings. The initiation of immunosuppressive therapy combined with UDCA was associated with a favorable clinical and biochemical response. At one-year follow-up, the patient remains clinically stable under azathioprine with minimal-dose corticosteroids, regular monitoring, and sustained biological improvement. Liver transplantation was discussed with the family as a potential option in case of disease progression.
